# IUC24380-82 Prophylactic antibiotics use in local anesthetic transperineal ultrasound-guided prostate biopsy: a retrospective observational study

**DOI:** 10.1093/oncolo/oyaf248.003

**Published:** 2025-08-29

**Authors:** K Rhodes, V Balasubaramaniam, A Agarwal, A Kailasa, K Donev

**Affiliations:** Betsi Cadwaladr Univeristy Health Board, Bangor, Wales, United Kingdom; Betsi Cadwaladr Univeristy Health Board, Bangor, Wales, United Kingdom; Betsi Cadwaladr Univeristy Health Board, Bangor, Wales, United Kingdom; Betsi Cadwaladr Univeristy Health Board, Bangor, Wales, United Kingdom; Betsi Cadwaladr Univeristy Health Board, Bangor, Wales, United Kingdom

## Abstract

**Background:**

Local anesthetic transperineal ultrasound-guided prostate biopsy (LATP) is increasingly preferred over transrectal ultrasound-guided biopsy due to a lower risk of post-procedural infection. However, the necessity of prophylactic antibiotic use in LATP remains uncertain. This study compares infection rates in patients undergoing LATP with and without prophylactic antibiotics. To evaluate whether the administration of prophylactic antibiotics before LATP reduces the incidence of post-procedural urinary tract infections (UTIs).

**Methods:**

A retrospective observational study was conducted involving 103 patients who underwent LATP under the care of a single urologist. The study participants were divided into 2 groups. In Group 1 (February-November 2022), 53 patients received 240 mg intravenous gentamicin before the procedure. In Group 2 (May 2024-Mar 2025), 50 patients received no prophylactic antibiotics. Patient demographics, pre- and post-procedure urine dipsticks, MSU results, and post-procedure UTI presentations were reviewed.

**Results:**

In Group 1, no patients developed UTIs following the biopsy, 2 experienced urinary retention but had negative MSUs. In Group 2, 3 patients (*n* = 3/50, 6%) developed post-procedural UTI symptoms and had positive MSU results and were treated with oral antibiotics. One of these had a known history of recurrent UTIs. No patients in either group developed sepsis or required hospital admission.

**Conclusions:**

While prophylactic antibiotics appear to eliminate post-procedure infections, the infection rate without antibiotics remains low and manageable. These findings align with emerging evidence questioning the necessity of prophylactic antibiotics in LATP. Given the risks of antibiotic use, such as resistance, renal toxicity, and cost, further prospective, large-scale trials are needed to inform future guidelines.

**Acknowledgments:**

We would like to thank the urology nursing staff and medical records department at Betsi Cadwaladr University Health Board for their valuable assistance in data collection and patient monitoring.

**References:**

1. Jacewicz M, Günzel K, Rud E, et al. Antibiotic prophylaxis versus no antibiotic prophylaxis in transperineal prostate biopsies (NORAPP): a randomised, open-label, non-inferiority trial. *Lancet Infect Dis*. 2022;22:1465-1471. [PMC][10.1016/S1473-3099(22)00373-5] [35839791]

2. Basourakos SP, Alshak MN, Lewicki PJ, et al. Role of prophylactic antibiotics in transperineal prostate biopsy: a systematic review and meta-analysis. *Eur Urol Open Sci*. 2022;37:53-63. [PMC][10.1016/j.euros.2022.01.001] [35243391]

